# DNA-Triggered Aggregation of Copper, Zinc Superoxide Dismutase in the Presence of Ascorbate

**DOI:** 10.1371/journal.pone.0012328

**Published:** 2010-08-20

**Authors:** Jun Yin, Si Hu, Wei Jiang, Liang Liu, Shemin Lan, Xuegang Song, Changlin Liu

**Affiliations:** Key Laboratory of Pesticide and Chemical Biology, Ministry of Education, and School of Chemistry, Central China Normal University, Wuhan, China; University of Canterbury, New Zealand

## Abstract

The oxidative damage hypothesis proposed for the function gain of copper, zinc superoxide dismutase (SOD1) maintains that both mutant and wild-type (WT) SOD1 catalyze reactions with abnormal substrates that damage cellular components critical for viability of the affected cells. However, whether the oxidative damage of SOD1 is involved in the formation of aggregates rich in SOD1 or not remains elusive. Here, we sought to explore the oxidative aggregation of WT SOD1 exposed to environments containing both ascorbate (Asc) and DNA under neutral conditions. The results showed that the WT SOD1 protein was oxidized in the presence of Asc. The oxidation results in the higher affinity of the modified protein for DNA than that of the unmodified protein. The oxidized SOD1 was observed to be more prone to aggregation than the WT SOD1, and the addition of DNA can significantly accelerate the oxidative aggregation. Moreover, a reasonable relationship can be found between the oxidation, increased hydrophobicity, and aggregation of SOD1 in the presence of DNA. The crucial step in aggregation is neutralization of the positive charges on some SOD1 surfaces by DNA binding. This study might be crucial for understanding molecular forces driving the protein aggregation.

## Introduction

Copper, zinc superoxide dismutase (SOD1) is a thermostable 32 kDa homodimeric enzyme that is abundant in eukaryotic cells and normally catalyzes the conversion of superoxide anion to peroxide hydrogen and dioxygen through cyclical reduction and oxidation of copper [1]. Its mutation and overexpression does not result in an increased protection, but rather create a variety of injurious effects, for example, involvement in the development of amyotrophic lateral sclerosis (ALS) by inducing motor neuron death [2]–[18].

Many hypotheses including aggregation and oxidative damage have been proposed to explain the toxicity of SOD1 [6], [9]. The aggregation hypothesis maintains that mutant proteins of SOD1 become misfolded and consequently oligomerize into high-molecular-weight species that end up in proteinaceous inclusions, and the oligomerized or aggregated proteins are, at some stage in their formation, selectively toxic to motor neurons [6]. Proteinaceous inclusions rich in SOD1 have been observed in tissues from ALS patients, ALS-SOD1 transgenic mice, and in cell culture models [7]–[27]. The visible aggregates or inclusions in SOD1-linked diseases may be one of the pathological hallmarks, and have been linked to any of the disruptions in cellular functions [6]–[12], [28], [29]. Polymorphous SOD1 aggregates have been shown to be generated *in vitro* from WT SOD1 and ALS-associated variants [30]–[33]. Moreover, it has been observed *in vitro* that the negatively charged species including DNA can induce or accelerate aggregation of SOD1 in different forms [33], [34], as observed for other proteins [35], [36].

The oxidative damage hypothesis maintains that SOD1 of various forms catalyzes reactions with abnormal substrates such as H_2_O_2_ that damage cellular components (including the protein itself) critical for viability of the affected cells [6], [37], [38]. The oxidative damage requires the involvement of the redox-active copper bound to SOD1 proteins. Most oxidative damage to SOD1 proteins *in vivo* was believed to occur by site-specific, metal-mediated mechanism in which a Fenton reaction of H_2_O_2_ with redox-active metals produces hydroxyl a radical which immediately oxidizes an amino acid residue in close proximity to the metal-binding sites and results in enzyme inactivation, oxidative modification of residues at or near the metal sites, and loss of metals, likely as observed *in vitro* [39], [40]. However, although there is increasing evidence indicating that elevated oxidative damage to SOD1 proteins are present in the tissue of ALS transgenic mice [37], it is not known currently if the oxidative modification is involved in the SOD1 aggregation *in vivo* because a recent study showed that the SOD1 isolated from the aggregates in several SOD1 transgenic mouse lines contained primarily full-length unmodified SOD1 proteins [41]. The *in vitro* oxidation leads to destabilization and dissociation of dimeric SOD1 at physiological concentrations (∼40 µM) prior to aggregation [40], [42]–[44]. The zinc-deficient WT SOD1 and some of its mutants should be prone to form visible aggregates *in vitro* upon treatment with ascorbate (ascorbic acid, Asc) or/and copper salt compared with the WT holoprotein [42], [45].

DNA has been observed to act as a template accelerating the aggregation of WT SOD1 *in vitro* under acidic conditions [33]. In order to support the conclusion, the present study *in vitro* examines the role that the physiologically relevant factors, including DNA and Asc within motor neurons and other cells [46], may accelerate the aggregation of WT SOD1 *in vitro* under neutral conditions. The presence of Asc has been observed to cause oxidative damage to WT SOD1 proteins under neutral conditions [42]. During probing the template effect of DNA in WT SOD1 aggregation *in vitro*, we have found that the DNA-mediated enrichment of and the acidic pH-triggered hydrophobic alteration of the SOD1 protein are two key steps for aggregation [33]. The aggregation of SOD1 is dramatically accelerated by DNA under acidic conditions, but not under neutral conditions since neutral pH cannot alter the hydrophobicity of WT SOD1. However, WT SOD1 can also be enriched by DNA through the electrostatic interactions between them under neutral conditions [47]. On the other hand, ALS-linked MT SOD1 proteins have been classified into two groups: WT-like and metal binding region mutants [7], [48]. The WT-like mutants have a stronger aggregation-prone propensity via the site-specific oxidation of the copper-coordinated histidine residues by the reactions of the copper with H_2_O_2_ [49], as compared with the metal binding region mutants [50], [51]. Different ALS-associated mutations promote SOD1 aggregation by essentially distinct pathways [12]. In essence, the WT SOD1 treated with Asc behaves like the WT-like mutants because Asc at pharmacologic concentrations generates H_2_O_2_ and Asc radicals through the metal-dependent reactions *in vivo* [52], resulting in the conversion of WT SOD1 into an oxidized forms that resembles the mutant proteins. The oxidized SOD1 proteins can acquire binding and toxic properties of ALS-associated mutants [3]. Since the aggregation-prone propensity of oxidized SOD1 proteins was observed *in vitro* to be weak [40], [42]–[44], we examined the accelerating effect of DNA that acts as a template in the oxidized WT SOD1 aggregation *in vitro*.

## Results

### Asc-Mediated Oxidation of SOD1 *in vitro*

Based on the previous reports [48], [49], [52], [53], the reactions of Asc or H_2_O_2_ with either copper on proteins (for example SOD1) or added redox metal ions in the presence of dioxygen are capable of producing reactive oxygen species (ROS) to oxidize SOD1 proteins under neutral conditions. Here, 0–8 mM Asc (normal concentrations in neurons and glial cells are 0.5–10 mM [46]) was used to lead to oxidation of WT SOD1 *in vitro* in this study.

**Figure 1:**
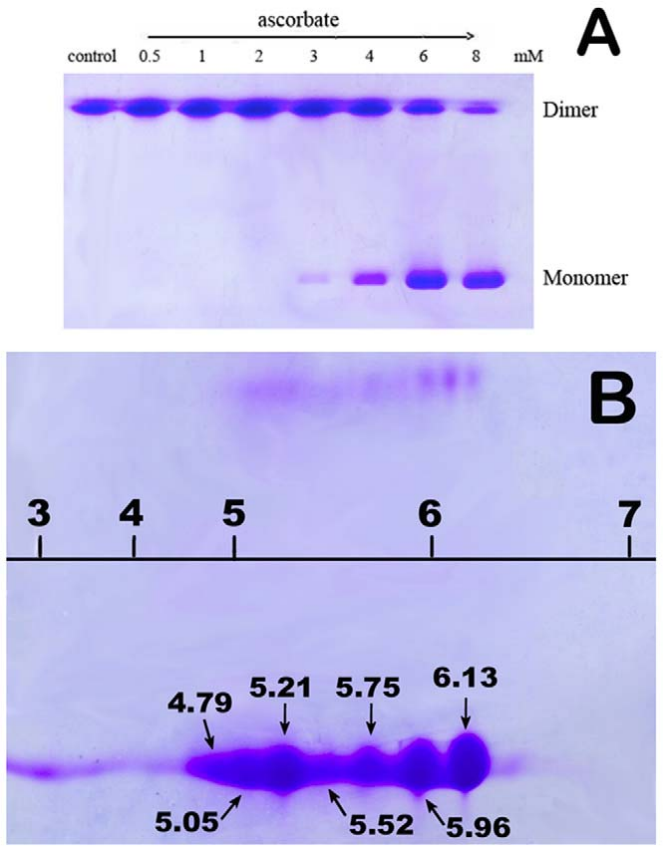
Observations of Asc-mediated WT SOD1 oxidation by one- and two-dimensional gel electrophoresis. A) Non-reducing SDS-PAGE gels for observation of Asc dose-dependent SOD1 oxidation. 10 µM SOD1 was incubated at 37°C for 2 h with 0–8 mM Asc. B) 2-DE gels for observation of p*I* and molecular mass of the SOD1 protein treated with Asc. 10 µM SOD1 was incubated with 4 mM Asc at 37°C for 2 h.

To investigate the oxidation of WT SOD1, first, the dependence of oxidation on Asc dose was examined with non-reducing sodium dodecyl sulfate-polyacrylamide gel electrophoresis (SDS-PAGE) at pH 7.4, because WT SOD1 is well known to be resistant to SDS. The non-reducing SDS-PAGE of SOD1 treated with Asc shows changes in stability upon oxidation. The observed SOD1 bands corresponding to dimeric and monomeric forms in gels (Figure 1A) showed that the SOD1 proteins treated with Asc were increasingly converted into monomers from dimers, indicating that the WT SOD1 dissociates into monomers upon oxidation. Obviously, any SOD1 aggregate was not observed in the SDS-PAGE gels, showing that the SOD1 aggregates, likely formed under the test conditions, were not SDS-resistant, as previously observed for the SOD1 proteins of other forms [54]–[56].

Then, to prove the results described above, we performed two-dimensional gel electrophoresis experiments on samples treated with 4 mM Asc. The SOD1 proteins were resolved by isoelectric focusing (IEF) on an immobilized pH gradient strip after Asc treatment. The results showed that many protein spots maintain the same molecular masses (∼16 and ∼32 kDa for monomer and dimer, respectively), but show different isoelectric points (∼ p*I* 4.8–6.1 for monomer, and p*I* 5.2–6.2 for dimer, Figure 1B). Here, the trace of dimeric species only observed in the two-dimensional gels is ascribed to the presence of 8 M urea in its loading buffer, which leads to dissociation of dimeric SOD1 into a monomeric form. The p*I* values found for the oxidized SOD1 proteins are well consistent with those found for the oxidatively modified isoforms of SOD1 (p*I* 6.3, 6.0, 5.7, and 5.0) extracted from Alzheimer and Parkinson disease brains [57]. Moreover, the p*I* for the protein spots of WT SOD1 treated by H_2_O_2_ was observed to be 5.3–5.6 due to the oxidation of its Cys111-SH to Cys111-SO_2/3_H [58]. Therefore, the differences in p*I* values might be caused by the different oxidized products of and the oxidative modification to Cys-SH groups on the SOD1 protein, as well as by the differences in the metallation extent of SOD1 protein.

### Changes in Hydrophobicity of Oxidized SOD1 Proteins in the Presence of DNA

The results delineated above reveal that the Asc-containing environment can lead to oxidation and dissociation of the dimeric protein. A question arises as to whether or not any change in the hydrophobicity or tertiary structure of the SOD1 protein appears upon oxidation, otherwise the oxidized SOD1 proteins cannot aggregate. Therefore, ANS (8-anilino-1-napthalene-sulfonic acid) dye binding experiments were performed on the Asc-treated SOD1 samples with and without DNA. ANS binding, which is a fluorescence probe that can indicate the disruption and formation of hydrophobic clusters in proteins, is proportional to hydrophobic surface area available for binding fluorophores.

**Figure 2:**
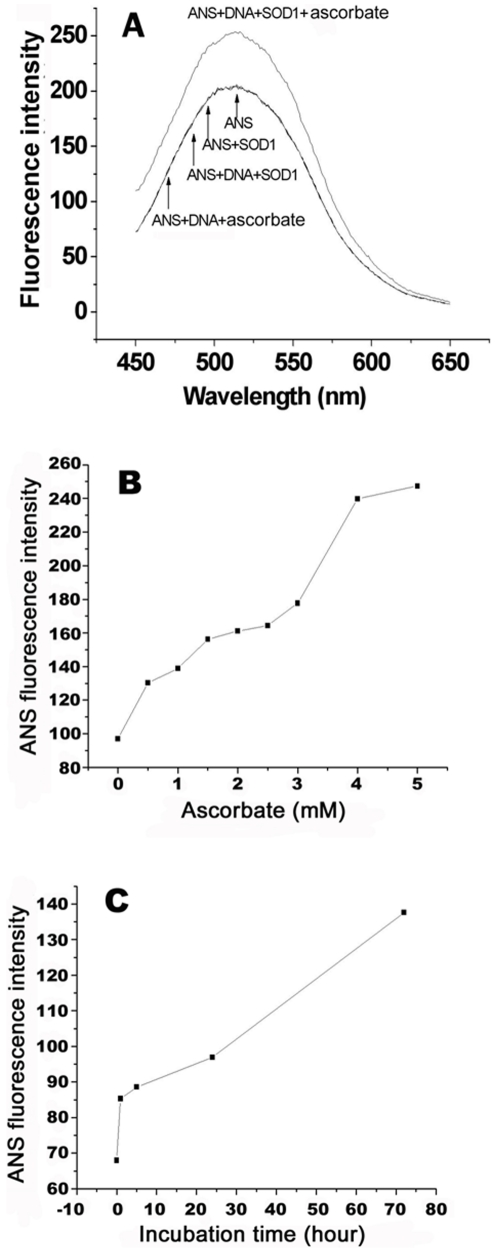
Comparison of ANS binding property of SOD1 in the presence and absence of both Asc and DNA in 20 mM Tris-HCl buffer (pH 7.4). A) Fluorescence spectra of 20 µM ANS added into the mixtures containing 4 µM SOD1, 4 µM SOD1 and 7.5 µM ctDNA, or 4 µM SOD1, 7.5 µM ctDNA and 2 mM Asc. B) Change in the emission of 20 µM ANS added into the mixtures containing 4 µM SOD1 and 7.5 µM ctDNA with Asc dose. C) Change in the emission of 20 µM ANS added into the mixtures containing 4 µM SOD1, 2 mM Asc, and 7.5 µM ctDNA with incubation time. Reactions were first incubated at 37°C for 2 h (A and B) or 0–72 h (C), and then re-incubated for 10 min at 37°C after addition of 20 µM ANS prior to measurement.

First, we determined fluorescence spectra of the ANS added into samples with and without Asc. The fluorescence spectra of the ANS added into the samples containing (1) SOD1, and (2) both SOD1 and ctDNA (Calf thymus DNA) at given levels were observed to be identical to that of the ANS alone in the same buffer. The addition of Asc into the SOD1 samples in the absence of DNA resulted in a slight increase in the ANS fluorescence (∼5%, Figure S1), whereas the addition of DNA led to at least 26% enhancement in emission of the ANS that was added into the mixture containing SOD1 and Asc under identical conditions (Figure 2A). These results indicated that (1) the WT SOD1 protein didn't show the ANS binding even in the presence of DNA, (2) ANS can bind to the oxidatively modified SOD1 proteins regardless of addition of DNA, and (3) the addition of DNA can further alter the hydrophobicity of oxidized SOD1 proteins. Therefore, the enhancement in the hydrophobicity of WT SOD1 is caused by the combination of Asc and DNA.

Then, we examined further the effect of Asc dose and incubation time on the ANS fluorescence. The ANS emission was observed to be progressively enhanced (Figure 2B) over the Asc dose range (0–4 mM) in the presence of DNA, showing that increasing doses of Asc led to exposure of more SOD1 hydrophobic surfaces. On the other hand, the ANS emission was also significantly enhanced over incubation time (0–72 h, Figure 2C), demonstrating that SOD1 binds more ANS molecules as the incubation period was prolonged. The hydrophobic enhancement of SOD1 protein caused by oxidation is consistent with that previously reported [40]. These facts indicate the exposure of more SOD1 hydrophobic surfaces that bind ANS regardless of presence of DNA.

### Binding of Oxidized SOD1 Proteins to DNA

**Figure 3:**
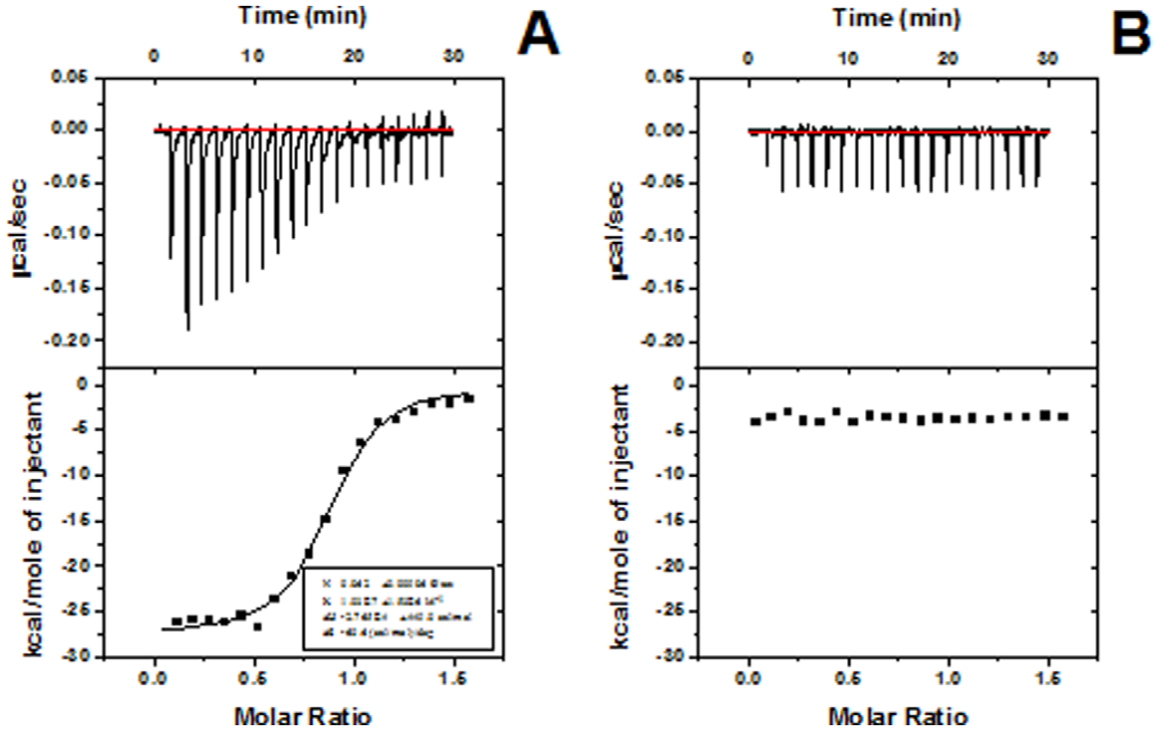
ITC analysis of oxidized SOD1 proteins binding to ssDNA. A) The upper panel shows the raw calorimetric data of the titration of ssDNA (40 µM) into WT SOD1 (5 µM) at 25°C in the 20 mM Tris-HCl buffer (pH 7.4) containing 4 mM Asc, and the lower panel shows the corresponding integrated injection heats, corrected for the heat of dilution. B) ITC data of the titration of ssDNA (40 µM) into SOD1 (5 µM) at 25°C in the 20 mM Tris-HCl buffer (pH 7.4) without Asc. All samples were incubated for 2 h at 37°C prior to titration under the identical conditions. The curve in the lower figure represents the best least-squares fits to the one-site binding model.

To understand the interactions between oxidized SOD1 proteins and DNA, we measured the binding parameters of the oxidized SOD1 to DNA with isothermal titration calorimetry (ITC). We have reported the equilibrium constant of ∼10 µM for the binding of WT SOD1 to double-stranded DNA (dsDNA) [47]. Here, we used 24-nt single-stranded DNA (ssDNA) instead of linear or supercoiled dsDNA to inhibit the dsDNA-triggered aggregation of oxidized SOD1 proteins. The length of 24-nt ssDNA should be long enough for the binding of the oxidized SOD1. In addition, utilization of the small ssDNA might be favorable for understanding the driving forces of SOD1 to DNA. Figure 3A shows the results from a typical ITC experiment in which ssDNA was titrated into the oxidized SOD1-containing solution. The data indicate that the binding of the oxidized SOD1 to DNA is an exothermic reaction with a large negative enthalpy change of –27.65±0.45 kcal/mol and a binding equilibrium constant of ∼100 nM, revealing that the strong binding between the oxidized SOD1 proteins and ssDNA is mainly determined by the electrostatic interactions. The binding stoichiometry was determined to be 0.86, indicating that (1) each ssDNA molecule may provide a single binding site for the oxidized SOD1, and (2) the oxidized SOD1 proteins bound to the ssDNA may not aggregate. However, an unfavorable entropy change was observed during the binding of the oxidized SOD1 to ssDNA, which might be attributed to formation of the ordered SOD1-DNA assemblies. We also measured the DNA binding property of WT SOD1 under the identical conditions, and the significant binding of WT SOD1 to ssDNA was not observed (Figure 3B), revealing that the interference from the unmodified SOD1 with the binding of modified SOD1 to ssDNA is negligible.

### Acceleration Roles of DNA in the Aggregation of Oxidized SOD1 Proteins

An *in vitro* study has indicated that either linear or supercoiled DNAs acting as a template can dramatically accelerate the SOD1 aggregation under acidic conditions [33]. The enhancement in the ANS binding indicates that the hydrophobicity of SOD1 was enhanced because of both oxidation and presence of DNA under neutral conditions (Figure 2A). Moreover, the oxidized SOD1 proteins were observed to have the higher affinity for DNA compared to the unmodified SOD1 (Figure 3A). Thus, we wonder if the DNA templates can promote aggregation of the oxidized SOD1 proteins under the identical conditions (pH 7.4).

**Figure 4:**
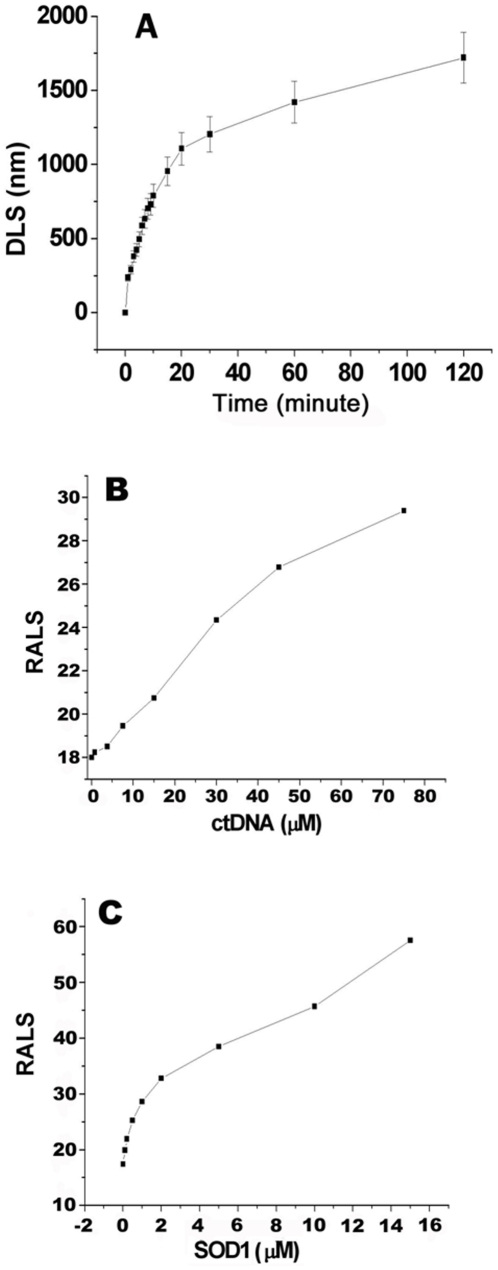
Dependences of DNA-triggered aggregation of oxidized SOD1 proteins on reaction conditions in 20 mM Tris-HCl buffer (pH 7.4). A) Reactions without stirring were incubated at 37°C for 0–120 min after addition of 7.5 µM ctDNA into the mixtures containing 4 µM SOD1 and 2 mM Asc. B) For DNA dose dependence, 0–75 µM ctDNA were added into the mixtures containing 4 µM SOD1 and 2 mM Asc were incubated at 37°C for 2 h. C) SOD1 dose dependence was observed by incubating reactions consisted of 0–16 µM SOD1, 2 mM Asc, and 7.5 µM ctDNA at 37°C for 2 h.

To examine the DNA-accelerated the aggregation of oxidized SOD1 proteins *in vitro*, time courses of reactions of the oxidized SOD1 proteins with DNA were monitored by dynamic light scattering (DLS). The same test conditions were maintained in controls containing Asc and either SOD1 or ctDNA. The DLS data showed that aggregates were immediately generated upon addition of ctDNA into the reactions containing both SOD1 and Asc without stirring (Figure 4A). The average hydrodynamic diameters of aggregates were dramatically increased to >1000 nm over 20 min. The aggregates maintain slow growth after incubation of 20 min. However, the significant aggregation of oxidized SOD1 proteins without DNA was not observed over incubation of 2 h, confirmed by right angle light scattering (RALS) measurements performed for the samples only containing either Asc and SOD1 or Asc and DNA (Figure S2). Indeed, the incubation of 24–48 h without DNA has been reported to be required to reach such the average diameter of 1000 nm for the aggregates of oxidized SOD1 [40], [42].

Then, the DNA dose dependence showed that the aggregation of oxidized SOD1 proteins without DNA occurs only when its concentration was ≥40 µM, as shown previously by RALS measurements [40], but aggregates were immediately observed to form from the reaction of 4 µM SOD1 and 2 mM Asc with 7.5 µM ctDNA (Figure 4B). In addition, we found by RALS measurements that the sizes of aggregates are almost linearly enhanced with ctDNA dose. Finally, the protein dose dependence observed by fixing doses of Asc and ctDNA showed that 0–2 µM SOD1 leads to the sharp increase, and larger SOD1 doses lead to the linear and slow increase in aggregate size (Figure 4C).

### Inhibition of the DNA-Triggered Aggregation of Oxidized SOD1 Proteins

Examining effects of the reaction conditions including ionic strength, guanidinium chloride (GdmCl) or chelating agents on the DNA-triggered aggregation of oxidized SOD1 proteins could be in favor of understanding the forces that drive the protein aggregation under neutral conditions.

**Figure 5:**
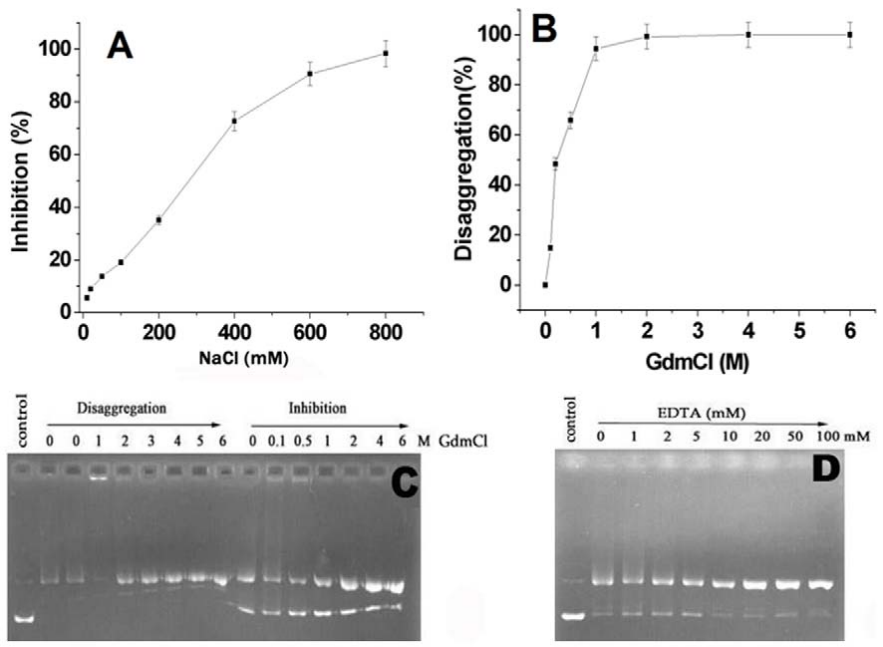
Inhibition of DNA-triggered aggregation of oxidized SOD1 proteins in 20 mM Tris-HCl buffer (pH 7.4). A) Effect of ionic strength was observed by incubating reactions containing 4 µM SOD1, 2 mM Asc, and 7.5 µM ctDNA at 37°C for 2 h in the presence of 0–800 mM NaCl. Here, the inhibition degree of aggregation is expressed by [(RALS)_0_– (RALS)_NaCl_]/(RALS)_0_×100%, (RALS)_0_ and (RALS)_NaCl_ represent RALS values in the absence and in the presence of NaCl at each concentration, respectively. B) The inhibitory effect of GdmCl was monitored by RALS measurements. Reactions containing 4 µM SOD1, 2 mM Asc, and 7.5 µM ctDNA were incubated at 37°C for 2 h in the presence of 0–6 M GdmCl. The disaggregation degree of aggregation is expressed as (A). C) The disaggregation and inhibition of aggregates was monitored by agarose gel electrophoresis. The aggregates were produced by incubating reactions containing 4 µM SOD1, 2 mM Asc, and 15 µM pBR322 DNA for 24 h at 37°C, and re-incubated for 1 min with 0–6 M GdmCl before loading onto gels. D) The inhibition of aggregation caused by EDTA was monitored by agarose gel electrophoresis. 4 µM SOD1 and 2 mM ascorbate were incubated for 2 h at 37°C with 15 µM pBR322 DNA in the presence of 0–100 mM EDTA before loading onto gels.

Firstly, probing the effect of ionic strength on aggregation can provide an insight into the nature of interactions between proteins and DNA. We have observed that interactions of SOD1 with DNA are markedly affected by ionic strength [33], [47]. Thus, the influence of NaCl on the DNA-triggered aggregation of oxidized SOD1 proteins was monitored by RALS. The data showed that the aggregation is gradually reduced as NaCl dose increased (Figure 5A). For example, 150 (physiological concentration) and 800 mM NaCl lead to reduction of 30% and 95% in aggregation degree, respectively. This result is in line with that obtained with ITC experiments, indicating that the binding of oxidized SOD1 proteins to DNA is driven by electrostatic interactions between them.

The inhibition behavior monitored by RALS indicated that 1 M GdmCl leads to reduction of 97% in aggregation extent (Figure 5B and 5C, left half). Moreover, the degradation experiments show that the degradation of aggregates occurs immediately upon addition of GdmCl, as indicated by appearance of the DNA bands with slight smear in agarose gels (Figure 5C, right half), well consistent with the fact that no SOD1 aggregate is observed in the SDS-PAGE gels, revealing that the SOD1 aggregates are not resistant to protein denaturants including GdmCl and SDS. The result suggests that the intermolecular hydrophobic forces of oxidized SOD1 proteins are one of the aggregation-driving forces. In addition, the inhibition experiments performed with EDTA showed the appearance of the clear DNA bands and almost complete inhibition of aggregation with increasing EDTA dose (Figure 5D). This result suggests that the oxidative aggregation must involve the copper on SOD1.

### Morphology of DNA-Triggered Oxidized SOD1 Aggregates

**Figure 6:**
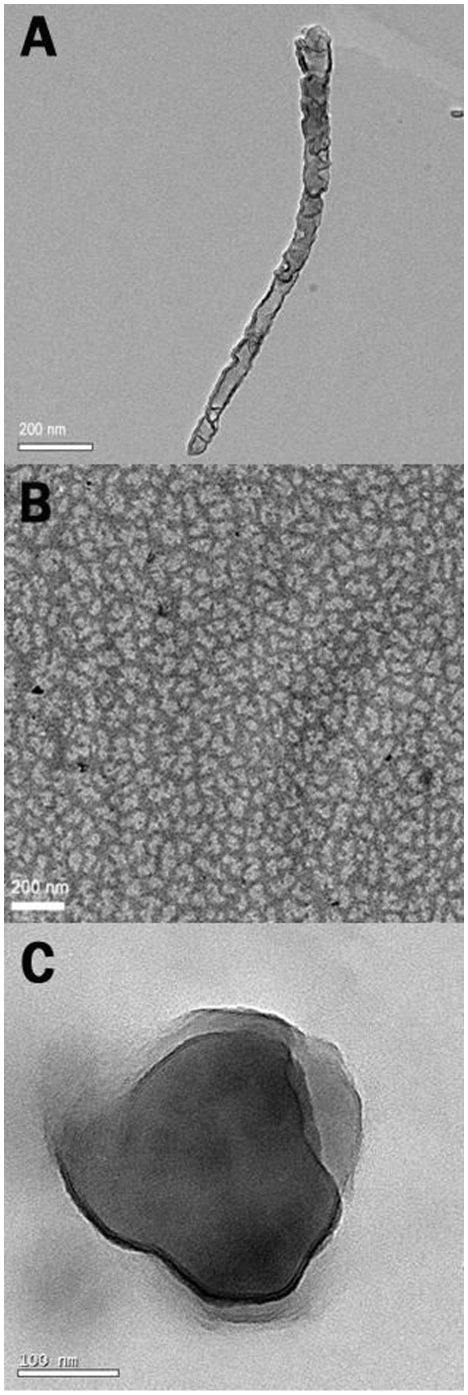
Visualization of aggregate and control samples under TEM. The controls were 7.5 µM λDNA (A) and 4 µM SOD1 (B) and incubated for 2 h at 37°C in the buffer (pH 7.4) containing 2 mM Asc prior to observation. C) Aggregate monomers were produced by incubating reactions containing 4 µM SOD1, 2 mM Asc and 7.5 µM λDNA for 2 h at 37°C.

In order to examine morphology of aggregates provided by the oxidized SOD1 proteins in the presence of DNA under neutral conditions, visualization of samples was performed under transmission electron microscopy (TEM) without staining. The DNA or SOD1 alone was first observed under TEM at pH 7.4 for comparison. The λDNA was observed to represent in a typical filament state (Figure 6A), and the SOD1 was in an amorphous state on the copper grid due to the formation of SOD1 aggregates caused by concentration of SOD1 during drying (Figure 6B).

One of the main aggregates observed under TEM in the λDNA-triggered aggregation containing 2 mM Asc under neutral conditions are the aggregate monomers consisted of a large number of oxidized SOD1 molecules and one λDNA molecule that is clearly visible here [33], [59]. This spherical aggregate monomer has an average diameter of 250–300 nm (Figure 6C). In contrast, although the SOD1 can associate with DNA under neutral conditions [47], any observable aggregation phenomenon under the TEM is not found without Asc, as indicated with DSL in examining the time courses of aggregation, revealing that Asc-induced SOD1 oxidation is a key factor in the enhanced aggregation propensity of WT SOD1. Moreover, incubating the reactions consisted of SOD1 and Asc does not produce any observable aggregate under the TEM in the absence of DNA. These facts indicate that the presence of DNA plays a critical role in Asc-induced oxidized SOD1 polymerization. In addition, the co-existence of DNA and proteins in the aggregates has been confirmed by fluorescent staining (ethidium bromide for DNA , thioflavin T/S for protein aggregates) experiments [33], [59].

## Discussion

The study presented here reported that three kinds of DNAs tested significantly accelerate the aggregation of oxidized SOD1 proteins *in vitro* under neutral conditions, as observed under acidic conditions [33]. The tested SOD1 proteins were isolated from human erythrocytes, and are highly relevant to aggregation, as reported by three independent groups [54]–[56]. Although SOD1 is known so far to be one of the most stable proteins, its conformation and stability can be influenced by mutation or oxidative modification, resulting in its monomerization, demetallization, increased susceptibility of disulfide bonds to reduction, reduction in repulsive charges, and proneness to aggregation [60], [61]. Here, we find that the exposure of WT SOD1 to the Asc-containing environment can also alter its stability, resulting in monomerization, and exposure of more hydrophobic surfaces.

Oxidation is one of the factors that cause SOD1 aggregation [40], [42]. Considering evidence of oxidative damage in sporadic ALS patients [62], [63] and abundance and ubiquity of the enzyme within cells [64], it seems plausible that SOD1 might constitute targets of oxidative damage in ALS. It has been reported that oxidative damage to SOD1 results in their dissociation into monomers and monomer destabilization, and aggregation *in vitro* [40]. The same phenomenon was observed in the Asc-induced SOD1 oxidation, but the concentrations of the SOD1 tested were nano- to micro-molar scale (Figure 4C), much less than its physiological level of ∼40 µM [40], and might fall in the concentration range of mutant SOD1 proteins. In the study, the enhanced hydrophobicity and gain of aggregation-prone propensity are found to be insufficient for the protein aggregation, if the protein does not reach the threshold concentration (40 µM here) required for aggregation. This is supported by the fact that the formation of oxidized SOD1 aggregates was not observed with the DLS and TEM in the range of concentrations tested in the absence of DNA-templated effect under identical conditions.

The ITC tests show that the binding of oxidized SOD1 proteins to DNA is enthalpy-driven and characterized by a high binding affinity. This observation leads us to suggest that the DNA binding neutralize the positive charges on SOD1 surfaces that prevent from aggregation, and significantly increases the local concentration of oxidized SOD1 proteins, resulting in a higher SOD1 concentration on the surface of DNA than that in bulk solution. Furthermore, it is observed that the more hydrophobic surfaces of oxidized SOD1 molecules became more solvent-accessible. Thus, DNA binding provides an avenue both to acquire a local and high concentration enough to facilitate the formation of aggregation-prone protein conformations and to enable the protein aggregation.

Two current apparently disparate hypotheses on the toxic gain of SOD1 functions may not be mutually exclusive when considering two finding: (1) there is a clear relationship between protein oxidation, hydrophobic enhancement, and DNA-triggered aggregation, and (2) SOD1 oxidation or exposure to acidic environments contributes to aggregation. Oxidation and acidic pH can alter protein structures and hydrophobicity, but it is uncertain if the repulsive charges on proteins can be reduced. According to the above observed DNA-triggered aggregation of the oxidized SOD1 proteins under neutral conditions, we found that the DNA binding-mediated reduction in the opposite charges on oxidized SOD1 protein surfaces provides an avenue to acquire the sufficient local concentrations to enable the SOD1 aggregation. Furthermore, reduction in the repulsive charge facilitates appearance of the intermolecular hydrophobic interactions of oxidized SOD1 proteins and formation of protein aggregates. Therefore, the accelerating effect of DNA in aggregation of the oxidized SOD1 proteins might be a consequence of three effects: reduced repulsively positive charges, altered hydrophobicity, and enhanced concentrations.

In summary, the reactions of Asc with redox metals on metalloproteins likely contribute to the redox state of cells that may play a role in protein aggregation. Using the important process of metalloenzyme-catalyzed oxidation, the aggregation of oxidized SOD1 proteins at nano- to micro-molar levels *in vitro* is accelerated by DNA. The process of DNA-triggered aggregation of oxidized SOD1 proteins was proposed as follows: association of the oxidized SOD1 proteins with DNA, reduction in the positive charges that might involve the prevention of SOD1 proteins from aggregation, enhancement in hydrophobicity, concentration of oxidized SOD1 proteins along DNA, association each other of DNA-bound oxidized SOD1 molecules, finally, formation of protein aggregates by the hydrophobic interactions. Recently, increasing evidence shows that indirect and direct interactions can occur between SOD1 and DNA or RNA both inside and outside cells [65]–[69]. Therefore, understanding the nucleic acid binding property of SOD1 might be in favor of understanding the intermolecular forces driving SOD1 aggregation, and provide a plausible explanation for the SOD1-induced ALS.

## Materials and Methods

### Materials

Calf thymus DNA (ctDNA), wild type SOD1 (isolated from human erythrocytes), ascorbate (Asc), ethidium bromide (EtBr), 8-anilino-1-naphthalene-sulfonic acid (ANS), and guanidinium chloride (GdmCl) were purchased from Sigma. The plasmid pBR322 DNA and bacteriophage λDNA were purchased from TaKaRa, the ssDNA (24-nt, 5′-GGTCGGAGTCAACGGATTTGGTCG-3′) was purchased from Invitrogen. Removal of impurities from SOD1 samples was monitored by liquid chromatography-electrospray ionization-mass spectrum on an Agilent1100 Cap LC/MSD XCT mass spectrometer. Dimeric SOD1 protein concentrations were estimated by the molar extinction at 280 nm of 10,800 M^−1^cm^−1^ [70]. Unless otherwise stated, DNA concentration was expressed in base pairs. All samples were prepared using distilled water that had been passed through a Millipore-Q ultrapurification system.

### Asc-Mediated SOD1 Oxidation in vitro

First, to examine the Asc dose dependence of SOD1 oxidation, 0–8 mM Asc was added into solutions containing 10 µM SOD1, and reactions were incubated at 37°C for 2 h in 20 mM Tris-HCl buffer (pH 7.4).

Non-reducing sodium dodecyl sulfate-polyacrylamide gel electrophoresis (SDS-PAGE) experiments were conducted on a 15% polyacrylamide resolving gel with a 5% polyacrylamide stacking gel. Samples were added 5× loading buffer without β-mercaptoethanol (β-ME). The gels were run at constant voltage of 80 V and stained by Coomassie Brilliant Blue R-250. Two-dimensional gel electrophoresis (2-DE) was carried out using an Ettan IPGphor 3 isoelectric focusing (IEF) unit and an Ettan DALTsix electrophoresis unit (GE Healthcare). IEF was performed with 7-cm Immobiline DryStrips (immobilized pH gradient strips, GE Healthcare) to generate a nonlinear pH gradient from 3 to 11. Proteins were visualized using Coomassie brilliant blue R-250 or Plusone silver staining kit (Amersham Biosciences). To minimize gel-to-gel variations, three 2-DE gels were run for the samples. The silver-stained gels were scanned and analyzed with ImageMaster 2D Platinum 6.0 software (GE Healthcare). Isoelectric points (p*I*) of spots were calculated according to the plot showing pH as a function of distance.

### Analysis of Hydrophobicity of Oxidized SOD1 Proteins in the Presence of DNA

To examine changes in hydrophobicity of oxidized SOD1 proteins, the fluorescence property of ANS bound to oxidized SOD1 proteins was examined on a Varian Cary Eclipse spectrofluorometer. Aliquots (300 µL) containing (1) 4 µM SOD1, (2) 7.5 µM ctDNA, (3) 4 µM SOD1 and 7.5 µM ctDNA, or (4) 4 µM SOD1, 7.5 µM ctDNA and 0–4 mM Asc were first incubated for 0–72 h at 37°C, and then re-incubated for 10 min with 20 µM ANS in 20 mM Tris-HCl buffer (pH 7.4), before measurement of fluorescence spectra between 450–650 nm (excitation at 365 nm).

### Binding Assay of Oxidized SOD1 Proteins to DNA

Isothermal titration calorimetry (ITC) was used to determine the binding parameters of oxidized SOD1 proteins to DNA and performed with a VP-ITC MicroCalorimeter. The samples were incubated for 2 h in the 4 mM Asc-containing buffer at 37°C and thoroughly degassed prior to titration. The sample cell was filled with 5 µM oxidized SOD1 or WT SOD1, and the syringe was filled with 40 µM ssDNA. The same buffer (20 mM Tris-HCl containing 4 mM Asc, pH 7.4) was used in both the syringe and the cell. The titration involved a single injection (1 µL) of and a series of injections (2 µL) of ssDNA solutions into the SOD1 solution. Analysis was carried out using Microcal Origin Software. Individual injections were integrated following manual adjustment of the baselines. Dilution and mixing heats were determined from separate control experiments or from the end point of the titration. This value was subtracted prior to curve fitting using a one-site model.

### DNA-Triggered Aggregation of Oxidized SOD1 Proteins in vitro

SOD1 at given doses was added into 20 mM Tris-HCl buffer (pH 7.4) containing 2–4 mM Asc. To trigger aggregation, ctDNA, λDNA or pBR322 DNA at given doses was added into the mixtures. The control reactions were each of reactants, mixtures of SOD1 with either Asc or DNA under identical conditions at each level used in aggregation reactions.

To examine the influence of reaction conditions on the oxidative aggregation, a series of experiments were carried out. Firstly, time courses of aggregation were observed in 0–120 min, for reaction consisting of 4 µM SOD1, 2 mM Asc and 7.5 µM λDNA. Then, 0–75 µM ctDNA was incubated with 4 µM SOD1, or 0–16 µM SOD1 was incubated with 7.5 µM ctDNA for 2 h in the buffer containing 2 mM Asc for observations of either DNA or SOD1 dose dependence. Finally, to recognize inhibition of SOD1 aggregation by ionic strength, GdmCl or EDTA, aggregation mixtures (4 µM SOD1, 2 mM Asc, 7.5 µM ctDNA) were incubated with 0–800 mM NaCl, 0–6 M GdmCl or 0–100 mM EDTA for 2 h. To examine disaggregation, 0–6 M GdmCl was added into SOD1 aggregates formed by incubation of the mixtures (4 µM SOD1, 2 mM Asc, and 7.5 µM ctDNA) for 24 h. The disaggregation reactions were incubated for 1 min at 37°C.

### Analysis of DNA-Triggered Aggregation of Oxidized SOD1 Proteins

The aggregation of oxidized SOD1 proteins with DNA was monitored with both right angle light scattering (RALS), and agarose gel electrophoresis. First, to determine the extent of SOD1 oxidative aggregation, RALS measurements were made at on the Cary Eclipse spectrofluorometer for each sample and each control (300 µL) according to the previously reported procedures [42]. Excitation and emission wavelengths were set all at 400 nm (band pass 4 nm) according to the average diameters of aggregates tested. Then, average hydrodynamic diameters of aggregates were evaluated by DLS [42]. DLS data were collected at 25°C on a HORIBA LB-550 dynamic light scattering particle size analyzer. The average diameters and their distributions were recorded for each sample and each control. Consecutive measurements (at least 200 times) were made with a cell of 2 mL for normalization analysis. Each spectrum represents an average of ten accumulations. For time courses, aliquots were taken from the aggregation mixtures at each designed time point, and DLS values were immediately recorded. In addition, DNA electrophoresis was conducted on a 0.8% agarose gel. The DNA gel bands were visualized by EtBr (0.5 µg/mL) staining.

### Visualization of the DNA-Triggered Aggregation of Oxidized SOD1 Proteins

Aggregates formed by the oxidized SOD1 proteins in the presence of DNA were visualized with a Tecnai G220 transmission electron microscope (TEM) [36]. Aliquots (5 µL) taken from incubation of a mixture of SOD1, λDNA or pBR322 DNA, and ascorbate at given concentrations for a given period at 37°C were adsorbed onto a thin carbon film-coated copper electron microscope grids (200-mesh), washed with the buffer, and air-dried for 5 min, followed by direct observations with TEM. The samples were not positively stained and directly imaged at 175 kV. Digital images of aggregation species were captured for final magnifications of 25–1,100,000×. All of these operations were also carried out for both SOD1 and DNA controls.
